# Effect of mold treatment by solvent on PDMS molding into nanoholes

**DOI:** 10.1186/1556-276X-8-394

**Published:** 2013-09-23

**Authors:** Celal Con, Bo Cui

**Affiliations:** 1 Department of Electrical and Computer Engineering, University of Waterloo, 200 University Ave. West, Waterloo, ON N2L 3G1, Canada; 2 Waterloo Institute for Nanotechnology (WIN), University of Waterloo, 200 University Ave. West, Waterloo, ON N2L 3G1, Canada

**Keywords:** Nanoimprint lithography, Molding, PDMS, Capillary filling, Surface energy

## Abstract

Polydimethylsiloxane (PDMS) is the most popular and versatile material for soft lithography due to its flexibility and easy fabrication by molding process. However, for nanoscale patterns, it is challenging to fill uncured PDMS into the holes or trenches on the master mold that is coated with a silane anti-adhesion layer needed for clean demolding. PDMS filling was previously found to be facilitated by diluting it with toluene or hexane, which was attributed to the great reduction of viscosity for diluted PDMS. Here, we suggest that the reason behind the improved filling for diluted PDMS is that the diluent solvent increases *in situ* the surface energy of the silane-treated mold and thus the wetting of PDMS to the mold surface. We treated the master mold surface (that was already coated with a silane anti-adhesion monolayer) with toluene or hexane, and found that the filling by undiluted PMDS into the nanoscale holes on the master mold was improved despite the high viscosity of the undiluted PDMS. A simple estimation based on capillary filing into a channel also gives a filling time on the millisecond scale, which implies that the viscosity of PMDS should not be the limiting factor. We achieved a hole filling down to sub-200-nm diameter that is smaller than those of the previous studies using regular Sylgard PDMS (not hard PDMS, Dow Corning Corporation, Midland, MI, USA). However, we are not able to explain using a simple argument based on wetting property why smaller, e.g., sub-100-nm holes, cannot be filled, for which we suggested a few possible factors for its explanation.

## Background

Nanoimprint lithography (NIL), which is not limited by light diffraction as in photolithography or charged beam scattering as in electron/ion beam lithography, is a low-cost and high-throughput process that offers ultrahigh resolution. The mold (or stamp) is typically fabricated from silicon for thermal NIL and quartz for UV-curing NIL, which are rigid and susceptible to breakage that reduces the lifetime of the mold and increases the cost of the process. A natural solution to this issue is a polymer mold material. Unfortunately, most common polymer materials (polymethyl methacrylate (PMMA), polystyrene, polycarbonate, etc.) are not suitable because they are incompatible with anti-adhesion surface treatment needed for clean demolding. The mold material has to either possess a low surface energy such as those containing fluorine or contain silicon whose surface can be converted into SiO_2_ upon oxygen plasma treatment (SiO_2_ is suitable for anti-adhesion surface treatment). The former group includes perfluoropolyethers [1] and Teflon AF 2400 (DuPont, Wilmington, DE, USA) [2], whereas the latter includes polydimethylsiloxane (PDMS) [3] and Si-containing UV-curable resist [4,5]. Another equally important property of the above materials is that the polymer mold can all be duplicated readily from a master mold as they are liquids in the uncured form.

Among the mold materials mentioned above, PDMS is the most popular and versatile mold material for nanoimprint and soft lithography because of its flexibility for conformal contact with non-planar surfaces, high UV transparency, low surface energy, high gas permeability, chemical inertness, and ease of handling. However, besides its low Young's modulus, it is found challenging to fill uncured PDMS into the nanoscale pattern on the master mold that is coated with an anti-adhesion monolayer needed for clean demolding. Previous studies have shown that PDMS filling into a nanoscale pattern can be facilitated by diluting it with toluene or hexane, which was attributed to the great reduction of viscosity for diluted PDMS [4,5]. However, if viscosity is the limiting factor, the hole filling depth should be increased with the filling time, which is not the case according to our experiment.

In addition, many reports including the above two are for PDMS filling into protruded features (e.g., an array of pillar) in the master mold that is easier when the pillars are well separated than filling into (recessed) holes. This is because for the application as nanoimprint mold, the hole pattern in PDMS (pillar in the master mold) is much more mechanically stable than the pillar pattern (hole in the master mold). For hole filling by PDMS, one study claimed filling of 100- to 200-nm diameter holes in porous alumina, but unfortunately, this claim was not supported by its experimental results [6]. Two other studies on PDMS filling into porous alumina also obtained very shallow and incomplete filling [7,8]. Another recent study showed complete filling into large 750-nm diameter holes in the silicon master mold coated with anti-adhesion layer [9]. In this study, we achieved a hole filling down to sub-200-nm diameter by additional solvent treatment of the mold that was already coated with an anti-adhesion monolayer. Our study suggests that the wetting properties between PDMS and mold are important for PDMS filling into the nanoscale pattern, and the improved filling by the diluted PDMS could be mainly due to the diluent toluene or hexane increasing *in situ* the surface energy of the anti-adhesion-treated mold, rather than due to the reduced viscosity of the diluted PDMS. As such, our study represents a significant step forward in understanding this very widely employed process. However, even taking into consideration of both viscosity and surface energy/wetting property, we are not able to explain why smaller holes cannot be filled. Further theoretical and experimental study is needed in order to elucidate the hole filling process by PDMS.

## Methods

Our silicon master mold contains arrays of nanoholes with diameters ranging from 1,000 nm down to 100 nm and depth close to 1,000 nm, and was fabricated by electron beam lithography and pattern transfer process. The hole array pattern was first exposed in ZEP-520A (Zeon Corporation, Tokyo, Japan) electron beam resist at 20 keV using Raith 150^TWO^ electron beam lithography system (Ronkonkoma, NY, USA). After development using pentyl acetate (Sigma-Aldrich, St. Louis, MO, USA) for 1 min at room temperature, the pattern was transferred into the Al hard mask layer using RIE with BCl_3_ gas. Next, the pattern was further transferred into the silicon wafer with Al as mask using Oxford Instruments ICP380 dry etching system (Abingdon, UK) with C_4_F_8_ and SF_6_ gases [10], followed by Al removal process. To facilitate demolding of the cured PDMS from the master mold without pattern fracturing, the surface of the silicon master mold was coated with a self-assembled monolayer of trichloro (1H,1H,2H,2H-perfluorooctyl)silane (FOTS, Sigma-Aldrich, St. Louis, MO, USA) in a vacuum chamber for 12 h at room temperature. The silane-treated mold was baked at 150°C for 20 min to further lower its surface energy [11].

For the molding process, PDMS (Sylgard 184, Dow Corning, Midland, MI, USA) was first mixed with its curing agent at the ratio of 10:1 and then casted onto the master mold. Next, we left the samples in a vacuum for approximately 2 h for degassing, during which time period the PDMS began to fill the holes on the master mold. Finally, the PDMS was cured at 100°C for 4 h on a hotplate in atmospheric condition and peeled off from the master mold. To study the effect of the additional solvent treatment of the silane-coated master mold on PDMS molding, right before (undiluted) PDMS casting, some master molds were dipped into toluene or hexane for 1 min and dried with nitrogen gun.

## Results

### Effect of solvent treatment on PDMS filling into nanoholes

**Figure 1:**
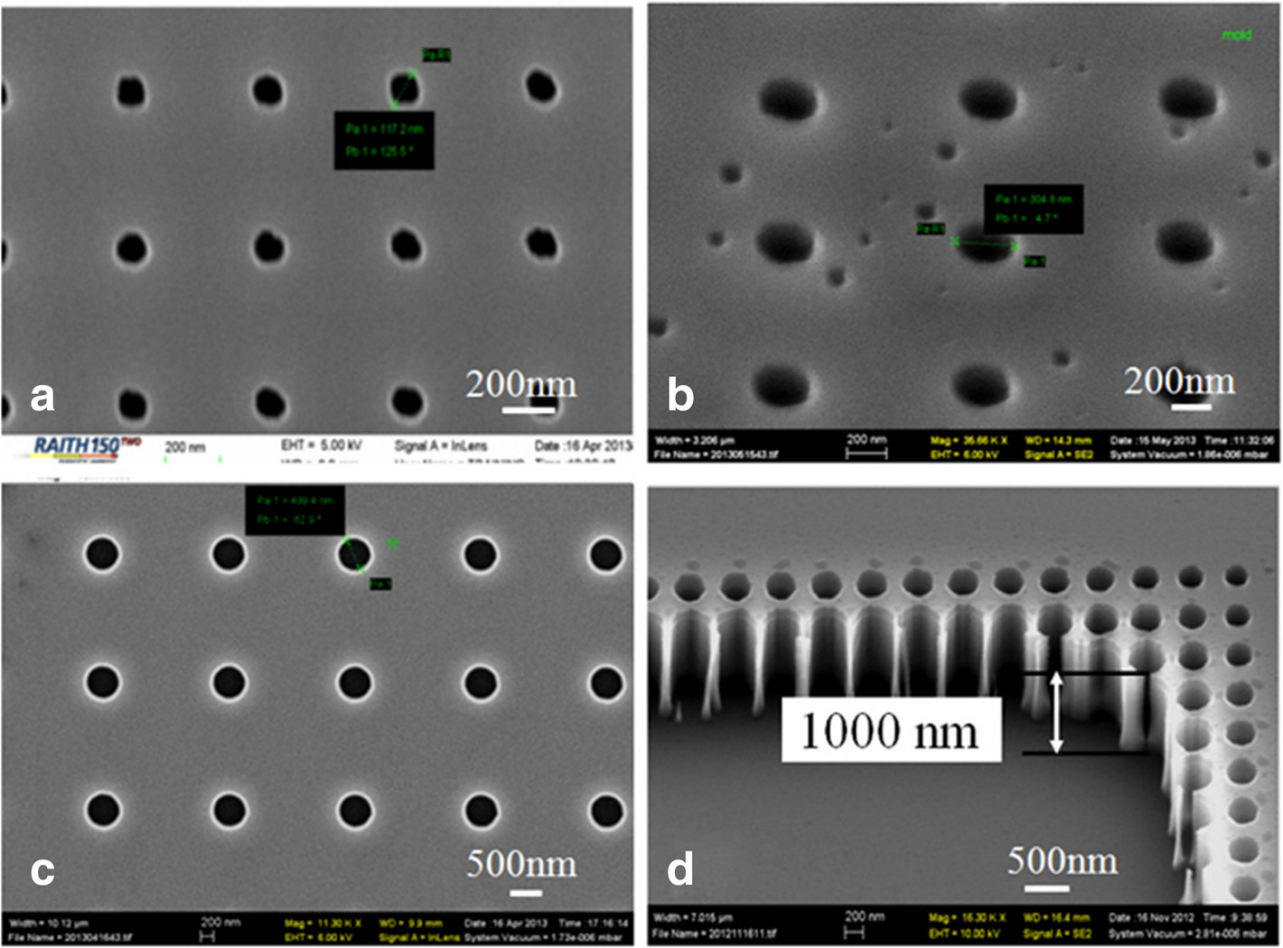
**SEM image of the hole array pattern in master mold (hole depth approximately 1,000 nm). (a)** Diameter 120 nm and array period 1,000 nm. **(b)** Diameter 300 nm and array period 1,000 nm. **(c)** Diameter 500 nm and array period 2,000 nm. **(d)** Cross-section near a large etched area, showing hole depth close to 1,000 nm. Samples were tilted 45° for SEM imaging.

Figure 1 shows the scanning electron microscopy (SEM) image of the master mold consisting of array of holes with various diameters. There are a total of ten different diameters in the mold; shown here are representative three with diameters of 500, 300, and 120 nm (smallest). Figure 1d is the cross-sectional view of the holes with diameter of 300 nm near a large etched area in order to reveal the etched profile, which shows a nearly vertical profile with depth close to 1,000 nm. However, the hole could be slightly shallower for smaller diameters due to the difficulty for etching species to diffuse into and for etching products to get out of the holes. Smaller holes are not necessary for the current study since, anyway, they could not be filled by the PDMS.

**Figure 2:**
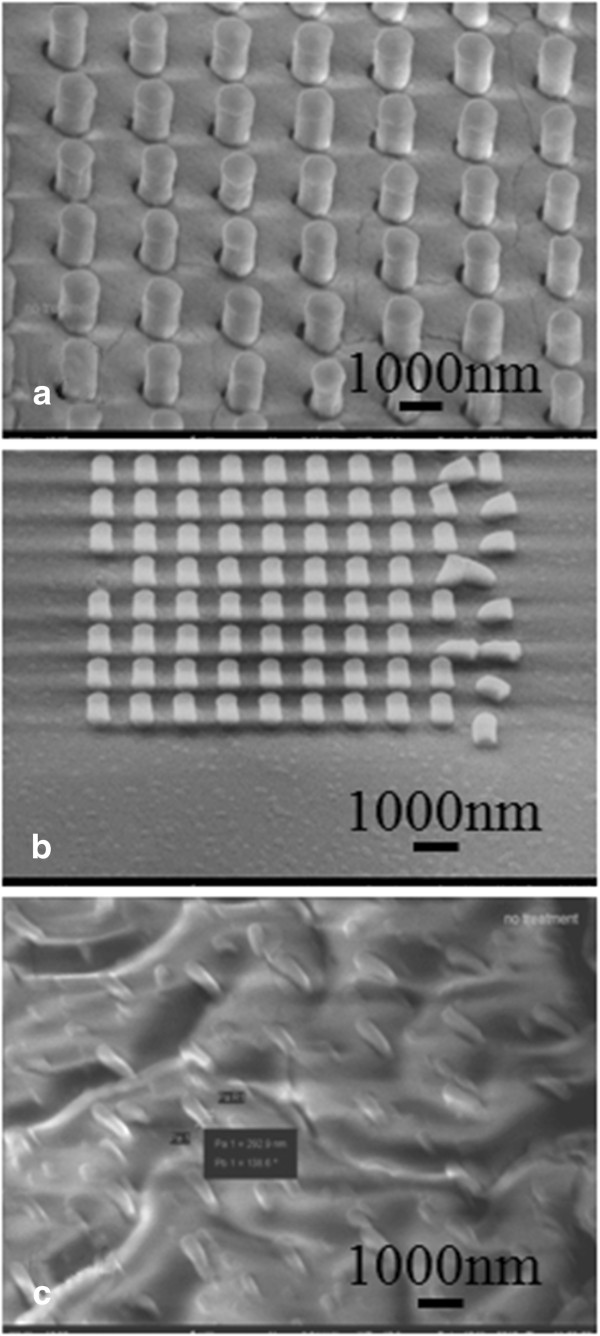
**SEM images of PDMS pillars.** The pillars were fabricated by molding with undiluted PDMS into the FOTS-treated master mold without additional solvent treatment. The pillar diameters are **(a)** 760 nm, **(b)** 500 nm, **(c)** 300 nm. Smaller holes were not filled. Pillar deformation and significant charging during SEM imaging are evident in **(c)**. Samples were tilted 45° for SEM imaging.

Figure 2 shows the filling of PDMS into the master mold treated with FOTS, but without any additional solvent treatment. For large diameters, the PDMS pillar array has a cylindrical shape matching the hole profile in the master mold. The smallest diameter that PDMS can successfully fill is about 300 nm, though for this diameter the pillars were deformed due to PDMS's low Young's modulus and the stress generated during demolding. Smaller holes were not fully filled with the PDMS, having a very short hemi-spherical ‘bump’ shape rather than a long cylindrical shape.

**Figure 3:**
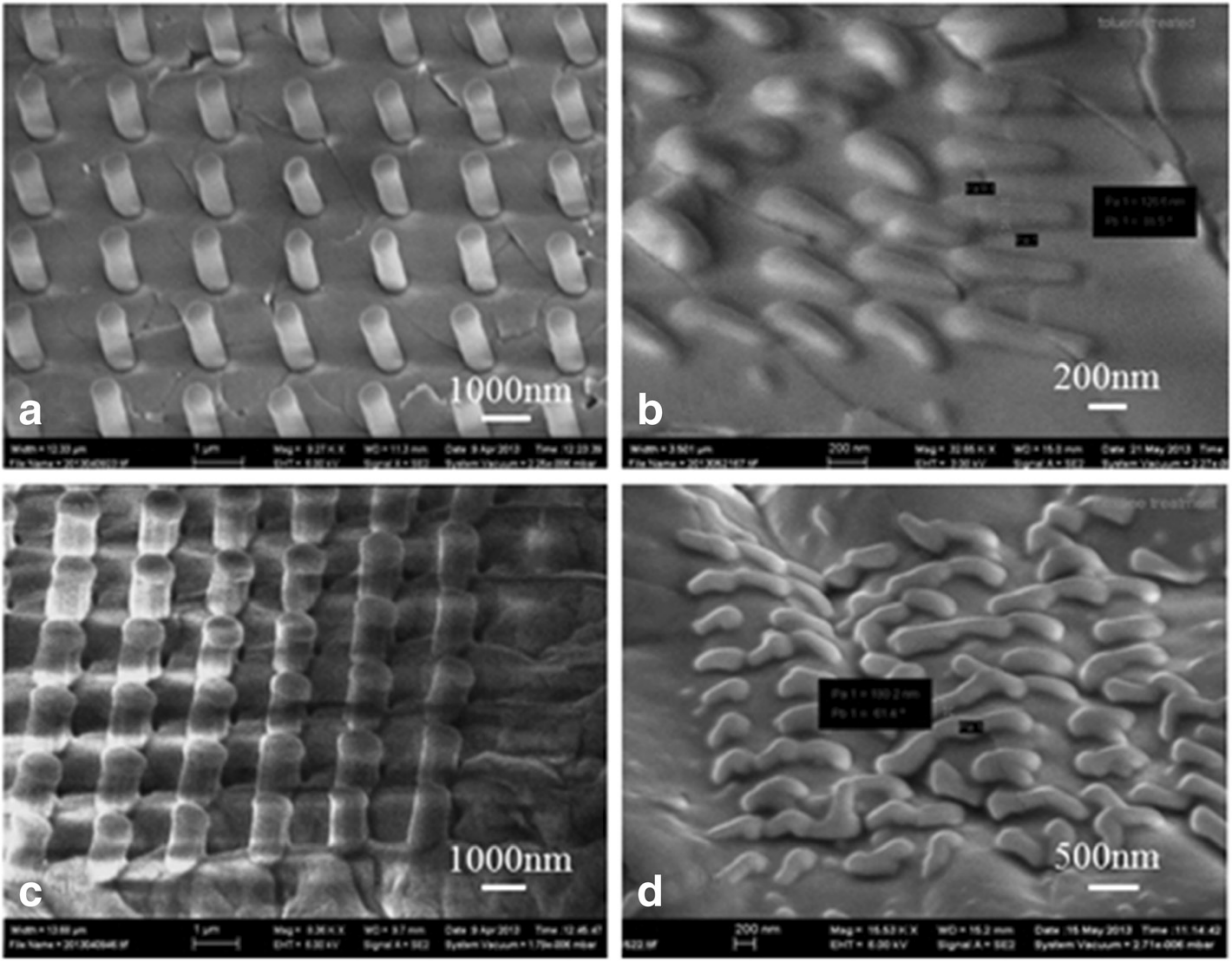
**SEM images of PDMS pillars molded into the toluene (a, b) or hexane (c, d) treated mold.** The pillar diameters are **(a)** 580 nm, **(b)** 150 nm (smaller holes not filled), **(c)** 820 nm, and **(d)** 180 nm (smaller holes not filled). Samples were tilted 45° for SEM imaging.

Figure 3 shows the PDMS pillar arrays molded into the master template treated with FOTS, with additional surface treatment using toluene or hexane solvent. The smallest PDMS pillar diameters are 150 and 180 nm for surface treated with toluene and hexane, respectively, which are both smaller than the diameter of the PDMS pillars (300-nm diameter) molded into a master template without solvent treatment. Though the improvement is not dramatic, this indicates that additional solvent surface treatment facilitated PDMS filling into FOTS anti-adhesion-treated master mold. It is possible that even though PDMS completely filled into the holes, we did not see PDMS pillars because they were broken during demolding. To verify this, we took SEM images of the master mold after PDMS filling and demolding, which revealed no PDMS left behind on the master mold.

## Discussion

In order to explain the enhanced PDMS filling by solvent surface treatment, we conducted water contact angle measurement on the three surfaces: FOTS-treated silicon, toluene- and FOTS-treated silicon, and hexane- and FOTS-treated silicon. The average measured contact angles are 107.8°, 104.1°, and 105.9° for the three surfaces, respectively. Though water contact angle is expected to differ greatly from PDMS contact angle as the two materials are very different, our measurement indicates an increase of surface energy upon additional solvent treatment, which could lead to an increase or even change of sign of capillary force that is proportional to *γ*_sa_ − *γ*_sl_ (here, *γ*_sa_ is the surface energy of the mold, and *γ*_sl_ is the interface energy of PDMS and the mold). This surface energy increase can be explained by the fact that significant percentage of FOTS is actually physically adsorbed (rather than chemically bonded) onto the mold surface and can thus be dissolved by the solvent, which results in the exposure of underneath bare silicon. More complete coverage by chemically bonded FOTS can be obtained through multi-cycle treatment, with each cycle consisting of FOTS treatment followed by dissolving physisorbed molecules.

Yang et al. has reported that water filling speed into a parylene microscale channel was increased by 2 orders by pretreating the channel with water, which was attributed to the water molecules' adsorption inside the channel and the resulted modification of parylene's surface energy [12]. As aforementioned, the PDMS filling into the silicon mold structures was improved by diluting it with a solvent such as toluene or hexane, which was attributed to the decrease of its viscosity [4]. Indeed, it is known that diluting PDMS drastically reduces its viscosity. For instance, its viscosity is reduced to 0.020 Pa · s by diluting it with heptane at 1:2 (PDMS/heptane) ratio [13], and for PDMS oligomers, the viscosity decreased from 0.362 to 0.050 Pa · s when diluted with toluene at 69% by weight [14]. It is fair to estimate that Sylgard 184 PDMS's viscosity is decreased by 1 order if diluted with toluene at 40 wt% (60% toluene, as is the case for [4]). Our study using undiluted PDMS with high viscosity suggests that the improved filling by dilution might be mainly due to the *in situ* modification of the mold surface energy and wetting property by the diluent.

The liquid filling speed into a *cylindrical* hole can be estimated following the derivation for *rectangular* holes in [12], as below.

• The capillary force applied on the fluid column: *F*_s_ = 2*πRγ*_la_ cos *θ*_c_

• The pulling pressure:

$P=\frac{2\mathrm{\pi R}\gamma_{\text{la}}\cos\theta_{c}}{\pi R^{2}}=\frac{2\gamma_{\text{la}}\cos\theta_{c}}{R}$

• The gradient of the pressure:

$-\frac{\mathrm{dP}}{\mathrm{dZ}}=\frac{P}{z}=\frac{2\gamma_{\text{la}}\cos\theta_{c}}{\mathrm{Rz}}$

• The velocity profile in a cylindrical hole:

$u\left(r,t\right)=2V_{\text{avg}}\left(1-\frac{r^{2}}{R^{2}}\right)$

• The average velocity:

$V_{\text{avg}}=\frac{\mathrm{dz}}{\mathrm{dt}}=-\frac{R^{2}}{8\mu }\left(\frac{\mathrm{dP}}{\mathrm{dz}}\right)=\frac{R^{2}}{8\mu }\times \frac{2\gamma_{\text{la}}\cos\theta_{c}}{\mathrm{Rz}}=\frac{R\gamma_{\text{la}}\cos\theta_{c}}{4\mu z}$

• Solving the differential equation:

$z=\sqrt{\frac{R\gamma_{\text{la}}\cos\theta_{c}}{2\mu }}t,\text{or}\;t=\frac{2\mu z^{2}}{R\gamma_{\text{la}}\cos\theta_{c}}$

Here, *μ* is the dynamic viscosity (3.9 Pa · s for Sylgard 184 PDMS), *z* is the filling depth (approximately 1,000 nm), *γ*_la_ is the PDMS surface tension, and *θ*_c_ is the contact angle (assume *γ*_la_ × cos*θ*_c_ approximately 0.001 N/m that is a very low value), and *R* is hole radius (approximately 100 nm), which leads to a filling time of only 0.078 s. The viscosity of the undiluted PDMS is roughly in the same order as that of the PMMA at *T*_g_ + 100°C (*T*_g_ is glass transition temperature) and is expected to be far lower than that of the polystyrene at 130°C (*T*_g_ + 25°C) due to the exponential relationship between viscosity and temperature, but the latter showed filling of 5-μm deep holes in porous alumina with diameter approximately 200 nm within 2 h [15]. Therefore, the poor filling of PDMS into the mold structure cannot be simply attributed to its low viscosity, and surface/interface property should play an equally important role as discussed above, as well as suggested by the previous study [14].

However, we are unable to explain why smaller holes such as 100- or 50-nm diameter were not filled with PDMS. In principle, as long as the PDMS ‘wets’ the mold, the filling time (∝1/R) should not increase drastically for smaller hole sizes (actually, in our experiment, the smaller holes could not be filled by increasing the filling time). Therefore, PDMS filling and curing into the nanoscale structures cannot be explained by the classical capillary liquid filling process, and other factors have to be taken into consideration, such as the following:

1) PDMS curing: volume shrinkage and curing time. The volume shrinkage of approximately 10% upon PDMS curing may pull out the PDMS structure that was already filled into the holes. For diluted PDMS, significant volume shrinkage occurs when solvent is evaporated, which may also pull out the filled PDMS. As for the curing time, to a certain extent, longer curing time is desirable since the filling will stop once PDMS was cured/hardened. The curing can be delayed by diluting PDMS with a solvent. In one study, a ‘modulator’ that lowers the cross-linking rate was introduced to PDMS and resulted in improved filling into 1D trenches [15]. However, the trench in that study is very shallow; thus, if PDMS can wet and fill the trench, it should fill it instantaneously. Therefore, the delay of curing might only help assure complete solvent evaporation before hardening.

2) The fact that the holes in our experiment are not open ended, and the trapped air could be compressed when the hole is being filled with PDMS from the top, which would in turn push the PDMS back. However, this factor should be insignificant as it was found that for smaller holes, the PDMS formed only very shallow bumps, so it did not fill the hole and thus the trapped air was not compressed. Moreover, the vacuum level (between 0.01 MPa and 10 Pa) was found unimportant for PDMS filling, though it affected the mechanical properties of the filled PDMS since the PDMS cured at poor vacuum was less dense due to trapped air and solvent molecule [16]. That is, the air at the dead end would dissolve in PDMS rather than get compressed since PDMS is air permeable.

3) Composition of the Sylgard 184 and its curing agent, which contains many additives. One important additive is silica nanoparticle filler for reinforcing purpose [17,18], which may block the hole when its size is not negligible compared to the hole diameter.

4) Size effect. The above derivation for capillary filling speed applies to large channels. For nanoscale holes, the filling mechanism is much more complicated. For example, the surface energy can differ significantly from macro-scale surface when the liquid pillar diameter is no longer orders larger than the range of van de Waals force, and the meniscus may be ‘pinned’ due to the abrupt change of surface topography or charges. In addition, at nanoscale, highly viscous fluid usually behaves like non-Newtonian fluid with much higher effective viscosity. Molecular dynamic simulation can be employed to better understand the PDMS filling mechanism.

Our current study only serves to suggest alternative roles of solvent in PDMS filling, and it cannot identify which factors play the most critical role in filling nanoscale holes. Systematic further study is needed to unambiguously elucidate the role of solvent for the hole filling by diluted PDMS, and why sub-100-nm holes are so difficult to fill. For instance, in order to focus on the effect of viscosity, pure PDMS with different molecular weights, thus very different viscosities, must be used to fill open-ended holes and examined in its liquid state (without curing). This will be studied and published elsewhere. From the point of view of practical application, PDMS filling into nanoscale holes can be improved by solvent dilution, surface treatment by solvent or surfactant other than FOTS such that the surface energy is just low enough for clean demolding, vacuum to drive off solvent and assure PDMS's mechanical property, and applied pressure that is the most effective approach [4].

## Conclusions

We, here, studied the effect of solvent treatment of the master mold surface (that was already coated with a silane anti-adhesion monolayer) on PDMS filling into nanoscale holes on the master mold. We achieved improved filling into holes with diameter down to sub-200 nm versus approximately 300 nm for master mold without this additional solvent surface treatment using toluene or hexane. Thus, we suggest that the improved filling by PDMS diluted with the same solvents is due to the *in situ* surface energy and wetting property modification by the solvent diluents, rather than due to the greatly reduced viscosity as proposed by previous studies. However, we are not able to explain why smaller holes (e.g., sub-100-nm diameter) cannot be filled, for which we suggested a few possible factors for its explanation.

## Competing interests

Both authors declare that they have no competing interests.

## Authors’ contributions

CC carried out the experiments and drafted the manuscript. BC guided the study and revised the manuscript. Both authors read and approved the final manuscript.

## Authors’ information

CC received his masters degree from the University of Waterloo in 2011 and is now continuing his PhD study at the same institute. BC is an Assistant Professor at the Department of Electrical and Computer Engineering, University of Waterloo.
